# A Radiotherapy Technique to Improve Dose Homogeneity Around Bone Prostheses

**DOI:** 10.1080/13577140410001679248

**Published:** 2004-03

**Authors:** M. V. Williams, N. G. Burnet, E. Sherwin, R. Kestelman, A. R. Geater, S. J. Thomas, C. B. Wilson

**Affiliations:** 1 Oncology Centre Addenbrooke's Hospital Hills Road Cambridge CB2 2QQ UK; 2 University of Cambridge Department of Oncology Addenbrooke's Hospital Hills Road Cambridge CB2 2QQ UK; 3 Medical Physics Department Addenbrooke's Hospital Hills Road Cambridge CB2 2QQ UK; 4 Oncology Centre Ipswich Hospital Heath Road Ipswich IP4 5PD UK

## Abstract

*Purpose*. Following limb conserving surgery for bone or soft tissue sarcoma, patients may require post-operative radiotherapy to minimise the risk of local recurrence. In such circumstances the metal prosthesis reduces the dose in its shadow by approximately 10% when using opposed fields. We describe a technique to boost the underdosed area to overcome this problem.

*Patients or subjects*. Seven sequential patients presenting between 1995 and 2001 had their treatment individualised because they had metal prosthesis in the treatment volume.

*Methods*. To improve the target dose homogeneity we used a custom-made keyhole cutout to boost the area in the shadow of the prosthesis. The degree of attenuation caused by the metal prosthesis was estimated and a boost dose calculated. Exit thermoluminescent dosimetry (TLD) was used to confirm the estimates made.

*Results and discussion*. Variation between patients was seen, demonstrating the need for exit TLD to individualise the treatment plan. The use of a boost field provides a method to overcome under-dosage in the shadow of a metal prosthesis. It improves dose homogeneity throughout the target volume and ensures adequate dose intensity around the prosthesis, the site most at risk of local recurrence.


Sarcoma, March 2004, VOL. 8, NO. 1, 37–42

Taylor & Francis healthsciences

ORIGINAL ARTICLE

### A radiotherapy technique to improve dose homogeneity around bone prostheses

M.V. WILLIAMS[1], N.G. BURNET[1,2], E. SHERWIN[1,*], R. KESTELMAN[3], A.R. GEATER[3], S.J. THOMAS[3] & C.B. WILSON[1]

[1]*Oncology Centre,* [2]*University of Cambridge Department of Oncology &* [3]*Medical Physics Department, Addenbrooke's Hospital, Hills Road, Cambridge CB2 2QQ, UK*

**Abstract**

*Purpose.* Following limb conserving surgery for bone or soft tissue sarcoma, patients may require post-operative radiotherapy to minimise the risk of local recurrence. In such circumstances the metal prosthesis reduces the dose in its shadow by approximately 10% when using opposed fields. We describe a technique to boost the underdosed area to overcome this problem.

*Patients or subjects.* Seven sequential patients presenting between 1995 and 2001 had their treatment individualised because they had metal prosthesis in the treatment volume.

*Methods.* To improve the target dose homogeneity we used a custom-made keyhole cutout to boost the area in the shadow of the prosthesis. The degree of attenuation caused by the metal prosthesis was estimated and a boost dose calculated. Exit thermoluminescent dosimetry (TLD) was used to confirm the estimates made.

*Results and discussion.* Variation between patients was seen, demonstrating the need for exit TLD to individualise the treatment plan. The use of a boost field provides a method to overcome under-dosage in the shadow of a metal prosthesis. It improves dose homogeneity throughout the target volume and ensures adequate dose intensity around the prosthesis, the site most at risk of local recurrence.

### Introduction

Limb conserving surgery for primary bone tumours is now a well-established practice and provides the best available functional outcome.[2,3,14,15] The patients require removal of the affected bone, with prosthetic replacement. Those at risk of local recurrence can be identified by a low tumour necrosis rate in the pathological specimen after primary chemotherapy[14] and close surgical excision margins[13] and pathological fracture.[1] In this setting, further local treatment with radiotherapy is often recommended. Very rarely, soft tissue sarcomas can involve the femur, requiring excision and prosthetic replacement of the bone. This clinical scenario typically also requires radiotherapy.

The principal component of prosthetic bone replacements is titanium, which has an atomic number ($Z=22$) close to that of calcium ($Z=20$), but a higher electron density. Its electron density relative to water is approximately 4, much greater than that of bone (typically 1.1–1.3). This adversely affects the depth–dose relationship in the tissues in its 'shadow'. Using conventional opposed fields to irradiate a limb there is an under-dose of approximately 10% in this region due to attenuation.[6] Metal hip prostheses are encountered regularly in radiotherapy planning; often avoidance is the preferred course of action, though sometimes the presence of the prosthesis is ignored where its impact on treatment is minimal. The reason for avoiding metal prostheses is 2-fold: firstly, it is usually impossible to determine exactly the elemental composition of the prosthesis, and, secondly, the majority of computer planning system algorithms cannot fully predict absorbed dose where metal is in the radiation field. In the treatment of osteosarcoma where resection is followed by insertion of a replacement,

*Current address: Oncology Centre, Ipswich Hospital, Heath Road, Ipswich IP4 5PD, UK.

Correspondence to: M.V. Williams, Oncology Centre, Addenbrooke's Hospital, Hills Road, Cambridge CB2 2QQ, UK. Tel.: +44-1223-274409; E-mail: michael.williams@addenbrookes.nhs.uk

1357-714X print/1369–1643 © 2004 Taylor & Francis Ltd
DOI: 10.1080/13577140410001679248

metal prosthesis bone, beams are arranged deliberately to treat the area around the prosthesis: avoidance is not an option.

We have developed a technique to overcome the problem of attenuation using a simple low melting point alloy (LMPA) cutout to compliment the prosthesis. A one-dimensional effective depth inhomogeneity calculation algorithm is used to estimate the dose reduction in the shadow of the prosthesis, which is then boosted with small fields shaped with individualised LMPA blocks. The technique described here has been used to improve dose homogeneity in a series of seven patients treated since 1994, all those who have required radiotherapy for sarcoma including a bone prosthesis.

Dosimetric studies on a phantom incorporating a prosthesis carried out in-house demonstrate that simple one-dimensional planning algorithms can reasonably predict the effect of a prosthesis, provided a value for the electron density is known.

### Method and materials

To use our technique, the planning target volume (PTV) and shielding blocks were drawn onto anterior and posterior localisation films and a representative set of patient outlines was taken to cover the longitudinal extent of the PTV. A cross-sectional impression of the prosthesis was transferred from film to outline. All contours were digitised into the planning system (TPS). In the absence of additional information, it was assumed that the prosthesis was titanium throughout, with a nominal electron density of 3.75 relative to water, derived from the CRC (Chemical Rubber Company) handbook.[18] In reality, prostheses are constructed from alloys and, more significantly, different parts of the prosthesis are made from different alloys.

Anterior and posterior fields were applied and, using the degree of attenuation predicted by the treatment planning system (TPS), the isodose distribution was optimised by altering beam weightings and normalisation point position (Fig. 1). Two opposing customised fields shaped using LMPA to match the prosthesis were applied to compensate for the dose ‘shadow’ behind the prosthesis with a weighting of approximately 10%: this improved the dose distribution (Fig. 2). In addition, simple compensation was added if required to account for the longitudinal change in patient contour. An estimate of the corrections required was calculated using the Addenbrooke’s radiotherapy treatment planning system (ARPS), using the one-dimensional effective depth inhomogeneity algorithm, rather than the modified Batho algorithm normally used for 6-MV calculations. Where possible, a narrow corridor of normal tissue, typically only skin and subcutaneous tissue, was left outside the edges of the fields. Minimal extra planning time is needed because of the simplicity of this technique.

The boost field shapes were digitised from localisation films (Fig. 3) and cut-out blocks were constructed from high-density LMPA. Positioning accuracy was checked at verification and subsequent portal images were acquired on treatment days. Set-up accuracy can be improved with electronic portal imaging, which allows positional adjustments to be made on-line (Fig. 4), and this has now been incorporated into our routine protocol. An accuracy of approximately 1 mm can be achieved with this technique. The time to set up and deliver this treatment is approximately the same as a four-field isocentric conformal plan using LMPA customised shielding blocks, and with an immobilisation device.

The differential dose volume histogram (DVH) (Fig. 5) demonstrates the improvement in dose

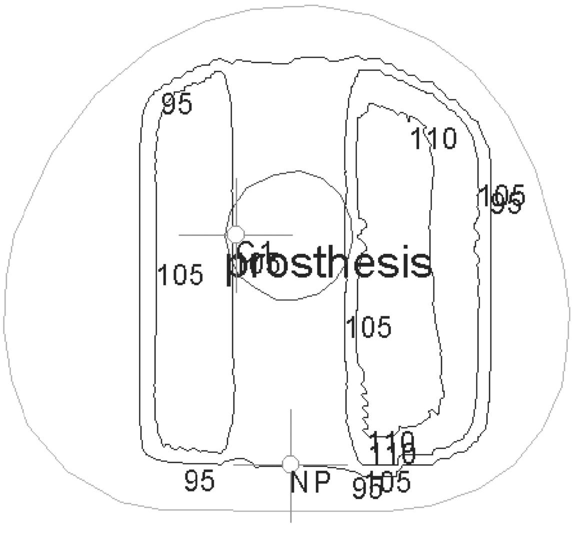

**Fig. 1.** *Central axis dose distribution; parallel opposed fields; prosthesis electron density 3.75. A large proportion of the PTV receives 110%.*

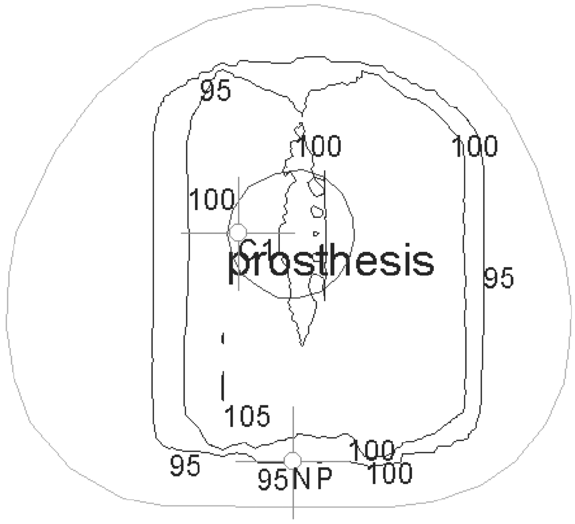

**Fig. 2.** *Plan including opposing boost fields applied to improve dose homogeneity.*

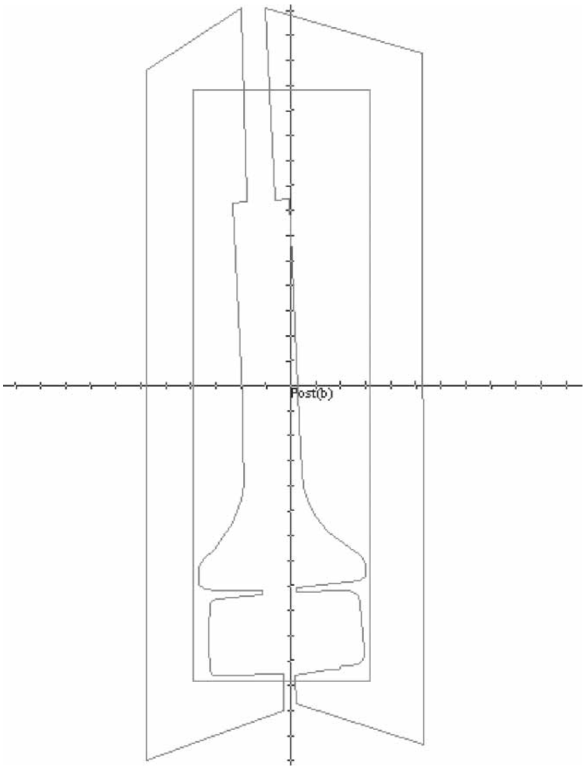

**Fig. 3.** *Boost field customised block template, designed to match the prosthesis' shape.*

homogeneity achieved by compensating for the attenuation by the prosthesis; the DVH approaches a single peak and there is less spread in dose. This DVH was derived from the central slice treatment plan of a recent patient; a representative volume was defined, avoiding the build-up region, for the purposes of comparison.

To distinguish between the more common prosthesis compositions of titanium alloy and steel/ cobal chrome (electron density ~ 7.0),[9,10,12,18] and to refine the boost field weighting, a second plan was produced to compare with *in vivo* thermoluminescent (TLD) exit dosimetry. This provides an independent means of determining whether the degree of attenuation estimated using the TPS is acceptable. To achieve this, all but one beam is deleted and 1 cm of bolus added to cover the exit contour. Dose points 1 cm apart underneath the bolus are recorded at the central axis and off axis where it is suspected that a change in prosthesis composition might occur. This arrangement is used on the first day of treatment and the measurements obtained are used to validate or adjust the boost dose contribution for the remaining fractions.

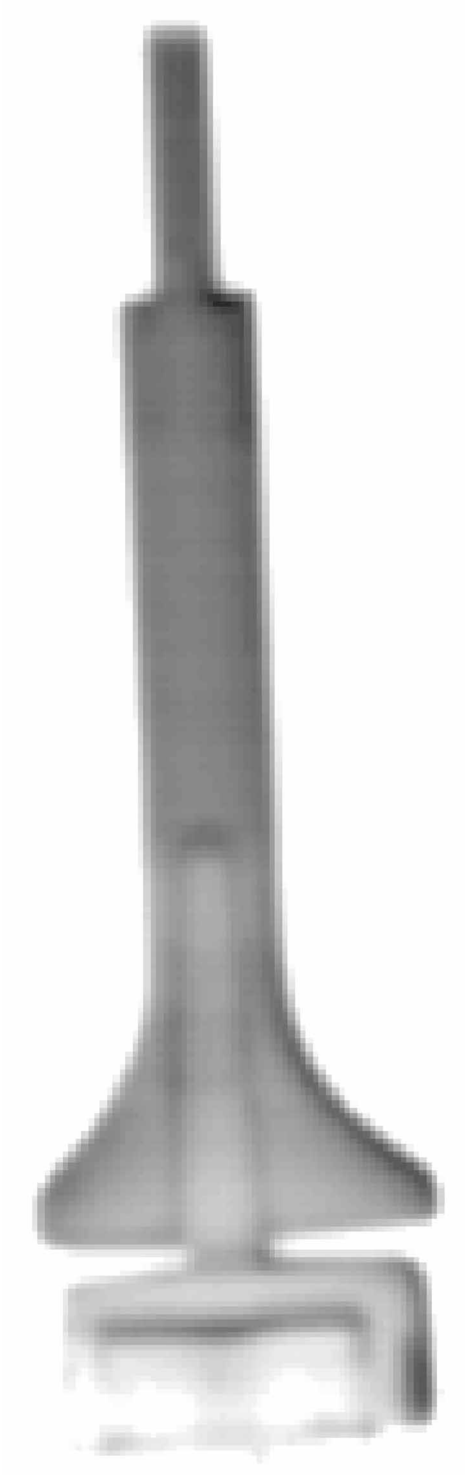

**Fig. 4.** *Daily portal image of a lower femur prosthesis to enable on-line positional adjustments. Darker areas indicate imperfect matching of the prosthesis and cut-out. In this case position is considered clinically satisfactory. The image also shows that the prosthesis has different components.*

### Results

To date seven patients have been treated using this technique. Their details and the reasons for post-operative radiotherapy are summarised in Table 1. With a median follow-up of 4 years (16 months–8 years) there has been one patient who has developed local recurrence, distal to the area irradiated, necessitating limb amputation and one patient who has died of metastatic disease with local control in the limb. A further patient has developed in-field recurrence and lung metastases; she remains well

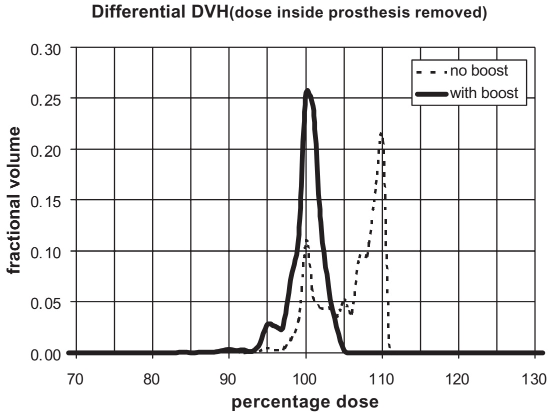

**Fig. 5.** *DVH demonstrates quality of dose homogeneity with and without boost fields.*

and disease-free 2 years after local excisions. The other four remain disease-free. Normal tissue effects have been modest.

Table 2 shows the monitor units required for the boost field (as a percentage of monitor units for the full fields) as calculated, and after correction for exit TLD. Whilst in most cases the calculated boost did not change significantly after TLD measurements, in one case (patient 5) it confirmed that there was a part of the prosthesis of considerably higher density (identified from localisation radiographs), requiring an extra boost. In patient 7, the overall density was higher than usual, requiring a greater boost. The fields in patient 3 were not parallel opposed, hence the difference between the fields.

### Discussion

Our technique uses customised prosthesis-shaped fields to boost the area shadowed by the prosthesis, and *in vivo* dosimetry to validate the attenuation estimate derived from the computer treatment planning system. The use of published electron density and the effective depth inhomogeneity correction algorithm is a good predictor of the degree of attenuation caused by a titanium alloy prosthesis; the accuracy of the estimate of electron density is not crucial due to the low boost field weightings involved. The dosimetric impact that loss of electronic equilibrium has at the tissue–prosthesis interface has also been investigated: our measurements show that dose enhancement due to backscatter upstream from the entry interface is greater than the dose reduction downstream from the exit interface, and this effect has a range of approximately 5 mm. Our findings are consistent with published data.[20] Tissue immediately adjacent to the prosthesis therefore receives an increased dose, but the volume irradiated is small and the maximum dose increase is estimated to be only 18% of local dose. We consider that this should be a beneficial effect, particularly since under-dose adjacent to the prosthesis is avoided, and the small volume receiving the higher dose is in the region where remaining tumour cells are most likely to be located.

Beyond the range of this interaction our technique improves dose homogeneity and eliminates the under-dose of 8–15% which would otherwise exist (Table 2).

We believe that this technique addresses an important issue, namely potential underdose in the shadow of the prosthesis in patients at high risk of local recurrence. In the seminal description of the correlation between intrinsic *in vitro* cellular radiosensitivity and clinical tumour control, Deacon *et al.*[5] assigned osteosarcoma cells to the most radioresistant category. This finding is consistent with clinical data from as far back as the original work by Cade, in the pre-chemotherapy era.[4] Some early dose–response studies performed on patients being treated with primary radiotherapy using the Cade technique found tumour sterilisation with doses of 70–90 Gy, but persistent tumour at doses less than or equal to 50 Gy.[4] Other clinical series from the pre-chemotherapy era have demonstrated poor local control with doses which would be considered standard today. In one series of 29 patients, no durable local control was achieved with doses in the range 45–60 Gy.[11] This suggests that osteosarcoma is relatively resistant to radiation. In an interesting analysis of dose–time dependence and response, Gaitan-Yanguas[8] described a range of doses from 20 Gy up to 100 Gy, and reported a dose response with doses above 60–70 Gy achieving a high probability of local control.

In the context of a multi-modality treatment programme, radiotherapy appears to have value for local control, and indeed survival, in standard doses.[13]

Other reports with small numbers of patients also suggest a role for radical radiotherapy as part of a multi-modality treatment programme.[16]

There is also evidence that local recurrence is associated with poorer survival from metastatic disease.[19] This is consistent with the notion of seeding from the recurrence, for which there is both laboratory[12] and clinical evidence.[7] This underlines the importance of maximising local control, both for local function and survival endpoints. These data suggest that for the high risk patients we have treated that it is important to maintain radiation dose intensity. Given the relatively radioresistant nature of this disease, maintenance of dose in the shadow of the prosthesis, a zone of high risk is likely to be of clinical value.

Because of the low numbers of patients who require post-operative radiotherapy in such circumstances, it will not be possible to evaluate the impact of this modification to standard treatment on local recurrence rate and overall survival. However, we believe it to be a valuable technique which is simple

**Table 1.** *Clinical details of seven patients treated*

| | Year | Diagnosis | Age (years) | Site | Pre-radiotherapy treatment | Indications for radiotherapy | Follow-up time | Current status |
|---|---|---|---|---|---|---|---|---|
| 1 | 1995 | Recurrent Haemangio-endothelioma | 21 | Right leg (distal femur/upper tibia) | Local excision | Recurrence after primary excision | 8 years | In field local control. Disease free after amputation for progression distal to RT field |
| 2 | 1998 | Osteosarcoma | 15 | Left proximal humerus | Pre-operative chemotherapy, excision, second-line chemotherapy | Low tumour necrosis rate, involved surgical resection margin | 5 years | In field recurrence and lung metastases resected at 3 years. Currently well and disease free |
| 3 | 1999 | Recurrent osteosarcoma | 17 | Right lower femur | Wide local excision of recurrence | Recurrence after primary excision | 4 years | Recurrence free |
| 4 | 1999 | High grade Leiomyosarcoma | 58 | Right quadriceps/femur | Pre-operative chemotherapy, excision | Femur encircled by soft tissue and removed at surgery | 4 years | Recurrence free |
| 5 | 2000 | Osteosarcoma | 18 | Right lower femur | Pre-operative chemotherapy, excision, second line chemotherapy | Low tumour necrosis rate, close resection margins, involvement of knee joint space | 2.5 years | Recurrence free |
| 6 | 2000 | Osteosarcoma | 21 | Right humerus | Pre-operative chemotherapy, excision | Pathological fracture at presentation | 16 months | No local recurrence. Died of metastatic disease |
| 7 | 2001 | High grade sarcoma rising in low grade osteosarcoma. | 34 | Right tibia | Excision, post-operative chemotherapy | Pathological fracture at presentation, close surgical excision margins | 2 years | Recurrence free |

**Table 2.** *Comparison of calculated and measured percentage dose reduction attributable to attenuation by the prosthesis*

| | % required for boost (calculated) | (after TLD measurement) |
|---|---|---|
| 1 | 12.4% | Not done |
| 2 | 10.1% | 10.1% |
| 3 | field 1: 14.8% | 14.1% |
| | field 2: 10.9% | 9.4% |
| 4 | 9.7% | 9.4% |
| 5 | 8.0% | part 8.0% |
| | | part 17.9% |
| 6 | 7.0% | 7.0% |
| 7 | 9.8% | 14.5% |

to instigate and does not add to the toxicity of treatment.

Further refinement of this technique may be possible using multileaf-collimation instead of alloy blocks, and intensity-modulated radiotherapy to account for the different attenuation properties of the prosthesis' composite parts.

### Acknowledgements

We thank Mrs Sally Ginn and Mrs Carol Faulkner for their expert typing of the manuscript. We would also like to thank the patient who donated his prosthesis after its replacement as this allowed us to undertake *in vitro* measurements.

### References

1. Abudu A, Sferopoulos NK, Tillman RM, Carter SR, Grimer RJ. The surgical treatment and outcome of pathological fractures in localised osteosarcoma. *J Bone Joint Surg Br* 1996; 78(5): 694–8.
2. Bacci G, Ferrari S, Bertoni F, *et al.* Long-term outcome for patients with nonmetastatic osteosarcoma of the extremity treated at The Instituto Ortopedico Rizzoli according to the Instituto Ortopedico Rizzoli/ Osteosarcomaa-2 protocol: an updated report. *J Clin Oncol* 2000: 18(24): 4016–27.
3. Bacci G, Picci P, Ruggieri P, *et al.* Primary chemotherapy and delayed surgery (neoadjuvant chemotherapy) for osteosarcoma extremities. The Instituto Rizzoli Experience in 127 patients treated preoperatively with methotrexate (high versus moderate doses) and intraarterial cisplatin. *Cancer* 1990; 65(11): 2539–53.
4. Cade S. Osteogenic sarcoma: a study based on 133 patients. *J R Coll Surg Edinburgh* 1955; 1: 79–111
5. Deacon J, Peckham MJ, Steel GG. The radioresponsiveness of human tumours and the initial slope of the cell survival curve. *Radiother Oncol* 1984; 2(4): 317–23.
6. Erlanson M, Franzen L, Henriksson R, Littbrand B, Lofroth PO. Planning of radiotherapy for patients with hip prosthesis. *Int J Oncol Biol Phys* 1991; 20: 1093–8.
7. Fuks Z, Leibel SA, Wallner KE, *et al.* The effect of local control on metastatic dissemination in carcinoma of the prostate: long-term results in patients treated with 125I implantation. *Int J Radiat Oncol Biol Phys* 1991; 21: 537–47.
8. Gaitan-Yanguas M. A study of the response of osteogenic sarcoma and adjacent normal tissues to radiation. *Int J Radiat Oncol Biol Phys* 1981; 7(5): 593–5.
9. Hazuka MB, Ibbot GS, Kinzie JJ. Hip prostheses during pelvic irradiation: effects and corrections. *Int J Oncol Biol Phys* 1988; 14: 1311–7.
10. Hudson FR, Crawley MT, Samarasekera M. Radiotherapy treatment planning for patients fitted with prostheses. *Br J Radiol* 1984; 57(679): 603–8.
11. Jenkin RD, Allt WE, Fitzpatrick PJ. Osteosarcoma. An assessment of management with particular reference to primary irradiation and selective delayed amputation. *Cancer* 1972; 30(2): 393–400.
12. Kaye GWC, Laby TH. *Tables of Physical and Chemical Constants.* 15th ed. New York: Longman; 1986.
13. Ozaki T, Flege S, Kevric M, Lindner N, Maas R, Delling G, Schwarz R, von Hochstetter AR, Salzer-Kuntschik M, Berdel WE, Jurgens H, Exner GU, Reichardt P, Mayer-Steinacker R, Ewerbeck V, Kotz R, Winkelmann W, Bielack SS. Osteosarcoma of the pelvis: experience of the Cooperative Osteosarcoma Study Group. *J Clin Oncol* 2003; 21(2): 334–41.
14. Provisor EJ, Ettinger LJ, Nachman JB, *et al.* Treatment of nonmetastatic osteosarcoma of the extremity with preoperative and postoperative chemotherapy: a report from the Children's Cancer Group. *J Clin Oncol* 1997; 15(1): 76–84.
15. Rosen G, Marcove RC, Caparros B, *et al.* Primary osteogenic sarcoma: the rationale for preoperative chemotherapy and delayed surgery. *Cancer* 1979; 43(6): 2163–77.
16. Schupak K. Bone sarcomas. In: Leibel SA, Phillips TL, eds. *Textbook of Radiation Oncology*. Philadelphia: WB Saunders, 1998.
17. Suit H. Potential for improving survival rates for the cancer patient by increasing the efficacy of treatment to the primary lesion. *Cancer* 1982; 50: 1227–34.
18. Weast, RC, ed. *CRC Handbook of Chemistry and Physics*. 58th ed. Ohio: CRC Press, 1977.
19. Weeden S, Grimer RJ, Cannon SR, Taminiau AH, Uscinska BM, European Osteosarcoma Intergroup. The effect of local recurrence on survival in resected osteosarcoma. *Eur J Cancer* 2001; 37(1): 39–46.
20. Dosimetric considerations for patients with hip prostheses undergoing pelvic irradiation. Report of the AAPM Radiation Therapy Committee Task Group 63. *Med Phys* 2003; 30(6): 1162–82.
